# Rasch validation of the Warwick-Edinburgh Mental Well-Being Scale (WEMWBS) in community-dwelling adults

**DOI:** 10.1186/s40359-023-01058-w

**Published:** 2023-02-17

**Authors:** Wei Deng, Sydney Carpentier, Jena Blackwood, Ann Van de Winckel

**Affiliations:** 1 Division of Rehabilitation Science, Department of Rehabilitation Medicine, Medical School, University of Minnesota, Minneapolis, MN USA; 2 Division of Physical Therapy, Division of Rehabilitation Science, Department of Rehabilitation Medicine, Medical School, University of Minnesota, 420 Delaware St SE (MMC 388), Rm 311, Minneapolis, MN 55455 USA

**Keywords:** Mental well-being, Healthy volunteers, Community, Rasch, Validation studies

## Abstract

**Background:**

With the ongoing global COVID-19 pandemic and the recent political divide in the United States (US), there is an urgent need to address the soaring mental well-being problems and promote positive well-being. The Warwick-Edinburgh Mental Well-Being Scale (WEMWBS) measures the positive aspects of mental health. Previous studies confirmed its construct validity, reliability, and unidimensionality with confirmatory factor analysis. Six studies have performed a Rasch analysis on the WEMWBS, and only one evaluated young adults in the US. The goal of our study is to use Rasch analysis to validate the WEMBS in a wider age group of community-dwelling adults in the US.

**Methods:**

We used the Rasch unidimensional measurement model 2030 software to evaluate item and person fit, targeting, person separation reliability (PSR), and differential item functioning (DIF) for sample sizes of at least 200 persons in each subgroup.

**Results:**

After deleting two items, the WEMBS analyzed in our 553 community-dwelling adults (average age 51.22 ± 17.18 years; 358 women) showed an excellent PSR = 0.91 as well as person and item fit, but the items are too easy for this population (person mean location = 2.17 ± 2.00). There was no DIF for sex, mental health, or practicing breathing exercises.

**Conclusions:**

The WEMWBS had good item and person fit but the targeting is off when used in community-dwelling adults in the US. Adding more difficult items might improve the targeting and capture a broader range of positive mental well-being.

**Supplementary Information:**

The online version contains supplementary material available at 10.1186/s40359-023-01058-w.

## Background

In recent years, the global COVID-19 pandemic has resulted in overworked healthcare workers, and many adults facing serious health problems, the death of loved ones, and fear of losing their job [1]. Coupled with a rise in violence caused by a political divide, the United States (US) has seen a 10% increase in the prevalence of adults with serious psychological distress in 2020 compared to 2018 [2]. Developing positive mental well-being and resilience has therefore become critically important.

Positive mental well-being relates to feelings of happiness and life satisfaction (i.e., hedonic aspects) as well as the purpose of life, full functioning of the person with a focus on realizing one’s own abilities and goals, being productive, coping with daily life stresses, and contributing to the community (i.e., eudaimonic aspects of life) [3, 4]. Purpose in life or meaning plays an important role in addressing stress, trauma, and adversity [1].

The Warwick-Edinburgh Mental Well-Being Scale (WEMWBS), developed by Tennant et al. [5], assesses positive mental health, covering both hedonic and eudaimonic aspects of positive well-being [3]. The internal consistency reflected by Cronbach's α was 0.89 and 0.91, in students and adults, respectively. Confirmatory factor analysis supported the unidimensionality of the scale [5]. WEMWBS has good high test–retest reliability (*r* = 0.83), good content validity, moderately high correlations with other mental health scales, and lower correlations with scales measuring overall health [6].

Aside from these psychometric properties obtained with classical test theory (CTT), six studies have investigated the structural validity of the WEMWBS in various countries with Rasch analysis. Rasch Measurement Theory is based on a predictive model stating that a person with a higher ability on a certain trait should have a higher probability of obtaining a higher score on the scale [6–9]. The Rasch analysis ranks the item difficulty hierarchically from easy to difficult on the same logit scale as the person’s ability [10–12]. The data have to meet the Rasch model requirement to form a valid measurement scale. In contrast, item response theory models are exploratory models aiming to describe the variance in the data. Rasch analysis also allows the transformation of an ordinal scale into an interval scale providing more measurement precision and information about measurement uncertainty along the scale [10–12].

The six studies that analyzed the WEMWBS with Rasch Measurement Theory obtained varied results in terms of targeting and the number of items that remained after the Rasch analysis was completed [6–9, 13, 14]. Of note, the data on the scale was acquired in different countries with possibly inherent differences in culture, which could at least partially explain this variation in results. Stewart-Brown et al. [6] analyzed data collected from adults in Scotland. They obtained item fit and good targeting (person mean location − 0.48 ± 1.22). Bartram et al. [8] analyzed data from veterinarians in the UK and presented a short 7-item unidimensional scale that fit the model, called the Short Warwick Edinburgh Mental Well-Being Scale (SWEMWBS). However, the items were too easy for this group (i.e., person mean location 1.15 ± 1.56). Melin et al. [13] also analyzed the SWEMBS in a Swedish population and reported the same issue with targeting. Houghton et al*.* [7] reported on a 10-item scale in adults in Western Australia with 3 misfitting items. Targeting was not reported. Wicaksono et al*.* [9] reported on the original 14-item scale with no misfitting items but the items were too easy for adults in Indonesia (i.e., person mean location 2.67 ± 1.56). To our knowledge, Marmara et al*.* [14] is the only study that investigated WEMWBS data in the United States of America (US) population as part of their sample collected in various countries (i.e., US, United Kingdom, Ireland, Australia, New Zealand, and Canada, total n = 394) with item response theory, using generalized partial credit model and graded response models. The sample included mostly younger adults ranging from 18 to 39 years with a mean of 27.54 ± 5.58 years old [14].

Therefore, the aim of this study is to assess the structural validity of the WEMWBS with Rasch in a wide age range of community-dwelling adults in the US. We will compare our findings with prior Rasch results.

## Methods

### Participants

For this cross-sectional study, we recruited participants at the Minnesota State Fair and Highland Fest and through volunteer sampling using research fliers and study postings on relevant websites. We also emailed the flier to volunteers who expressed interest in research from the Brain Body Mind Lab at the University of Minnesota. Recruitment occurred from September 27, 2017, till August 12, 2020. We included adults between 18 and 99 years of age, English speaking, and able to consent. All community-dwelling adults completed an anonymous questionnaire and thus gave verbal informed consent after acknowledging having read the consent form. The participants were subsequently quizzed on the comprehension of the content of the consent form through the University of California, San Diego Brief Assessment of Capacity to Consent (UBACC) [15]. The WEMWBS questionnaire was completed either on a tablet (at Minnesota State Fair and Highland Fest) or their personal computer at home. All completed questionnaires were stored on the secure UMN REDCap platform. The study was approved by the University of Minnesota's Institutional Review Board (IRB# STUDY00005849) and they were in accordance with the Declaration of Helsinki.

### Main outcome measures

The Warwick questionnaire covers positive aspects of mental health. All 14 items have a scoring range from “0-None of the time” to “4-All of the time”. A higher score on each item indicates a more positive attitude towards life. We collected demographic information, and whether participants currently practiced mindfulness, breathing exercises, or body awareness exercises (e.g., Yoga, Qigong, Pilates). We inquired whether they had current pain conditions or current mental health conditions.

### Statistical analysis

Following the recently accepted guidelines for reporting Rasch analyses, we report on structural validity and unidimensionality with overall fit, item and person fit, examining the presence of reversed thresholds, person separation reliability (PSR), differential item functioning (DIF), principal components analysis of residuals (PCAR), targeting, floor, and ceiling effect [11, 12].

Unidimensionality refers to the fact that all items should measure one construct. Item-trait interaction measures the overall fit of the scale to the Rasch model using Chi-square statistics. A non-significant *p* value indicates the scale fits the model. However, a large sample size can influence this *p* value even when all items fit the model. Person and item fit are reported through Chi-square statistics. Residuals greater than 2.5 or smaller than 2.5 indicate item redundancy and item misfit, respectively [10]. Item fit analysis takes into account Bonferroni corrections for multiple comparisons [16]. Disordered thresholds of scoring categories can be corrected by merging adjacent categories to improve fit to the model [10, 16].

PSR evaluates how well individuals or groups of different ability levels can be distinguished from each other [17]. DIF occurs when the hierarchies of items are significantly different between two sample subgroups (e.g., men vs. women) for sample sizes of at least 200 persons in each subgroup. DIF is calculated with an analysis of variance (ANOVA) with Bonferroni correction [16]. We calculated DIF for sex (men; women), mental health conditions (yes; no), and current practice of breathing exercises (yes; no) based on Marmara et al.’s [14] finding regarding different item invariance in sex as well as the importance of considering psychological diagnostics. Furthermore, we were interested in seeing whether people who include breathing exercises in their daily life as a lifestyle choice would score better on the WEMWBS, and whether those that self-report on mental health conditions would score lower on the WEMWBS.

Further evidence of unidimensionality can be evaluated with the Principal Component Analysis of Residuals (PCAR), which refers to the extent to which covariance in the residuals is random and not explained underlying constructs than the one that is being measured [10, 18]. In that case, the expected eigenvalue is less than 2, and the percent variance explained by the first component is less than 10%. If those criteria are not met, then dependent *t*-tests between the 2 subsets of items with positive and negative loadings on the first residual component are performed. We would confirm unidimensionality if less than 5% of these tests are significant. A scale is well-targeted when the person mean location is between − 0.5 and 0.5 logits and thus matching the average difficulty of the items (by default the item mean location is 0 ± 1 logits) [19]. Floor and ceiling effects need to be reported when at least 15% of the sample obtains a minimum or maximum score on the scale [20]. Residual correlations, as a measure of local item dependence**,** examines whether two items have more in common with each other than with the whole scale. Local item dependence is reported when two items have a correlation at least 0.2 above the average residual item correlation [21]. We used the Partial Credit Model and analyzed the data with Rasch Unidimensional Measurement Model (RUMM) 2030 software (RUMM Laboratory, Perth, WA, Australia).

## Results

We recruited 553 community-dwelling adults. The demographic, clinical, and behavioral characteristics of all participants are presented in Table 1.

**Table 1:** Demographic, clinical, and behavioral characteristics of participants by group

	Community-dwelling adults in the US (n = 533)
Age (years, Mean ± SD)	51.22 ± 17.18
Sex (n)	
Male	194
Female	358
Other	1
Ethnicity (n)	
Hispanic or Latino	8
Not Hispanic or Latino	545
Racial background (n)	
American Indian or Alaska Native	2
Asian	30
Black/African American	11
Hawaiian or other Pacific Islander	0
White	491
Multi-racial	10
Other	9
Pain (n)	113
Mental health conditions (n)	230
Current breathing exercise (n)	225
Current mindfulness exercise (n)	181
Current body awareness training (n)	180

### Rasch measurement theory

The iteration analysis displays the step-by-step approach taken for the Rasch analysis (Additional file 1). The main results are described below.

**Fig. 1:**
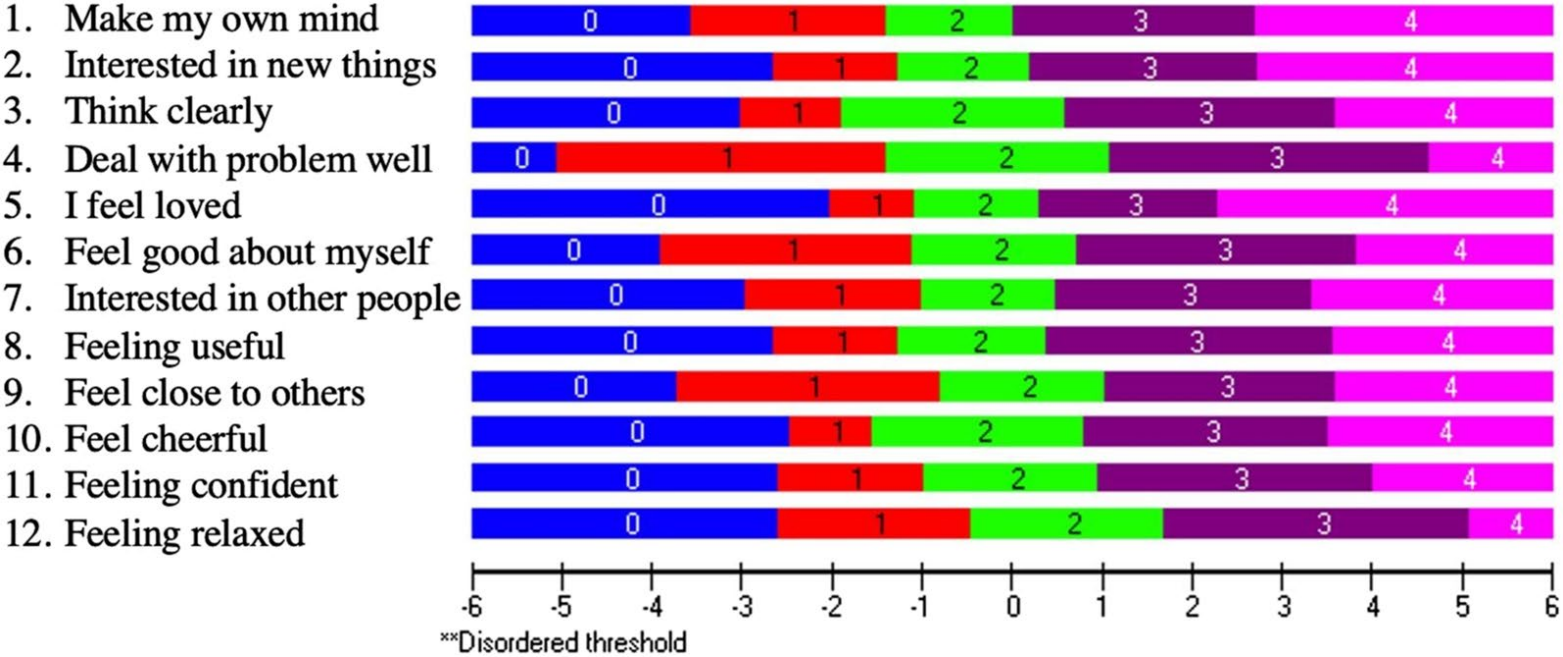
Item threshold map in community-dwelling adults in the US. The item threshold map shows the hierarchy of the item difficulty levels, with the easiest item on top (item 11 “*I've been able to make up my own mind about things*”) and the hardest item at the bottom (item 3 “*I've been feeling relaxed*”). The horizontal logit ruler demonstrates the person's ability level of positive mental health from low ability on the left to high ability on the right

**Fig. 2:**
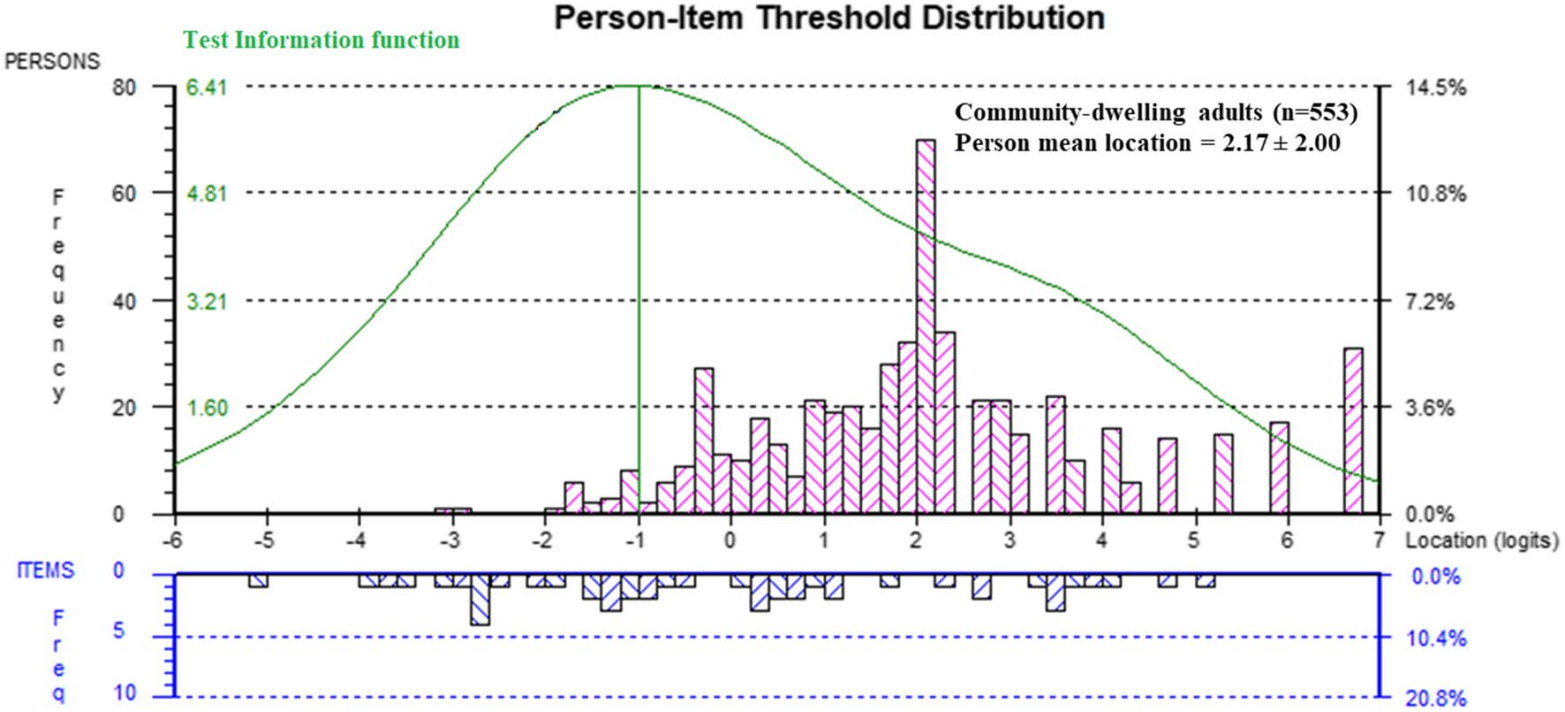
Person-item threshold distribution in community-dwelling adults in the US. The horizontal logit ruler represents both item difficulty and person ability. The pink histograms show the frequencies of the person's ability level in terms of positive mental well-being. A higher logit value indicates the person has a higher level of positive mental well-being. The blue histograms represent the frequencies of item difficulty level, and the items are organized from the easiest on the left to the hardest on the right. The green curve is showing the test information function, displaying where most information about the persons is provided and are inverse functions of the measurement standard errors (SE)

For our first analysis in community-dwelling Americans, none of the 14 items displayed disordered thresholds. Two items were misfitting: item 1 “*I have been feeling optimistic about the future*” and item 5 “*I have had energy to spare*.” After deleting items 1 and 5, all items fit the model and only 2.71% of persons were misfitting. The hierarchy of the item difficulty is presented in Fig. 1, with the easiest items starting at the top and the hardest items at the bottom. The item logit location and fit statistics are presented in Table 2; the item threshold locations are presented in the Additional file 2; and the frequency of scoring category responses per item in the Additional file 3*.* There was no floor or ceiling effect, but the person mean location ± standard deviation was 2.17 ± 2.00 logits, meaning that the items were too easy for this population (Fig. 2). The PSR was 0.91, indicating that we can distinguish individuals with different positive mental health levels. However, caution needs to be applied as the estimate of PSR could be misleading when the scale is badly targeted, such as is the case here. PCAR’s eigenvalue was 2.04 with 16.97% variance explained by the first component. The paired *t*-test revealed that 7.59% of the persons had significantly different logit locations on the two subtests. These results presume the existence of two dimensions in the scale. No DIF was found. No consequential local item dependence was found.

**Table 2:** Item fit statistics of the WEMWBS in community-dwelling adults in the US

Item number	Item descriptions	Item location (logits)	SE	Fit residuals	*p* value
Item 11	I've been able to make up my own mind about things	− 0.56	0.07	2.13	0.05
Item 13	I've been interested in new things	− 0.25	0.07	− 0.84	0.21
Item 7	I've been thinking clearly	− 0.18	0.08	− 0.37	0.84
Item 6	I've been dealing with problems well	− 0.18	0.08	− 0.13	0.84
Item 12	I've been feeling loved	− 0.12	0.07	1.18	0.04
Item 8	I've been feeling good about myself	− 0.11	0.07	− 4.92	0.003
Item 4	I've been feeling interested in other people	− 0.03	0.07	1.24	0.21
Item 2	I've been feeling useful	0.02	0.07	1.90	0.17
Item 9	I've been feeling close to other people	0.03	0.07	− 0.83	0.71
Item 14	I've been feeling cheerful	0.08	0.07	− 4.65	0.04
Item 10	I've been feeling confident	0.35	0.07	− 5.47	0.002
Item 3	I've been feeling relaxed	0.93	0.07	2.72	0.01

*SE* standard error

We also tested if the fit and unidimensionality would improve if we deleted items to match the 7-item SWEMWBS mentioned in previous studies. There were no misfitting items. The PCAR’s eigenvalue was 1.86 with 26.53% variance explained by the first component. The paired *t*-test revealed that 8.50% of the person logit pairs had significantly different locations. Additionally, the PSR dropped from 0.92 to 0.82, which would only allow researchers and clinicians to make group decisions, rather than individual decision-making [22, 23]. Moreover, the items were still too easy (person mean location 1.88 ± 1.71). We therefore do not recommend using the 7-items scale for clinical use. We recommend that the targeting first be solved before it can be used in the clinic or for research and, therefore, we do not provide a revised scoring sheet or score-to-measure table for the 12-item revised scale.

## Discussion

The aim of this study was to investigate the structural validity of the WEMWBS in a wide age range of community-dwelling adults living in the US. The WEMWBS showed good item and person fit. The main problem was the targeting, demonstrating that the items were too easy. These findings were consistent with the findings in all other studies that reported on person mean locations with Rasch analysis, except for Stewart-Brown et al. [6], who reported good targeting [7–9, 13, 14]. Of note, similar to Melin et al*.* [13], there are gaps in the item threshold attribute values especially at the right-hand side of the scale (Fig. 2), where more difficult items are, accompanied by larger measurement uncertainties, indicated by the green curve in Fig. 2). The best measurement region is situated around − 1 logits, which is more at the lower well-being end of the scale. There are 75 participants between the logits − 2 and 0 (i.e., around the point/area of the maximum reliability).

Of note, item fit in the community-dwelling adult group was obtained after deleting misfitting items 1 and 5. Deleting item 5 “*I’ve had energy to spare*” was consistent with earlier studies [6–8]. In Houghton et al*.* [7], item 5 was deleted because DIF was identified for age, while item 5 demonstrated misfit in both Stewart-Brown et al*.* [6] and Bartram et al*.* [8]. Item 1 “*I have been feeling optimistic about the future*” was maintained in prior studies. During a qualitative study on item comprehension of the WEMWBS, a focus group in Pakistan noticed difficulties in answering “Feeling optimistic about the future”, because there is no translation for “optimistic” in Pashtun [24]. Teenagers in Northern Ireland also expressed difficulty in answering item 1 [25]. We did not perform a qualitative analysis after this study and thus were unable to identify the reason for misfit in our US sample. The PCAR analysis pointed to two underlying dimensions underneath positive mental health. The items that loaded positively on the first principal component—items 4 “*I have been feeling interested in other people”*, 9 *“I have been feeling close to other people”*, and 12 *“I have been feeling loved”*—all seemed to point to positive feelings regarding interpersonal relationships. The items that loaded negatively on the first principal component seem more related to eudaimonic aspects of life in terms of a person feeling productive regarding their goals and feeling in control of their lives. These were items 6 *“I have been dealing with problems well”*, 7 *“I have been thinking clearly”*, and 8 *“I have been feeling good about myself”*.

To expand on Melin et al.’s statement that item 2 “*I’ve been feeling useful*” may have a different significance and importance in relation to culture because the item attribute value is relatively higher in Sweden (located at 0.21 logits) than in the UK (located at 0.00 logits) or Australia (located at − 0.14 logits), our results show that the location of this item in our US cohort (located at 0.02 logits) is similar to the one in the UK (Table 3) [7, 8, 13]. Item 2 in the Swedish SWEMWBS analysis has the second highest location (6th out of 7 items), while the US, Australian, and UK cohorts have item 2 respectively, as the 8th location out of 12 items (5th highest); 5th item location of 10 items, and 3rd item location out of 7 items [7, 8, 13].

**Table 3:** Item locations for our US cohort, compared to previously published Swedish, UK, and Australian cohorts

Item	US cohort (12 items)		Swedish cohort [13] (7 items)		UK cohort [8] (7 items)		Australian cohort [7] (10 items)	
	Location	SE	Location	SE	Location	SE	Location	SE
11. I've been able to make up my own mind about things	− 0.56	0.07	− 0.57	0.01	− 1.07	0.04	− 0.85	0.04
13. I've been interested in new things	− 0.25	0.07					0.08	0.03
7. I've been thinking clearly	− 0.18	0.08	− 0.47	0.02	− 0.66	0.04	− 0.48	0.04
6. I've been dealing with problems well	− 0.18	0.08	− 0.02	0.02	0.08	0.04	− 0.19	0.03
12. I've been feeling loved	− 0.12	0.07					− 0.42	0.03
8. I've been feeling good about myself	− 0.11	0.07						
4. I've been feeling interested in other people	− 0.03	0.07						
2. I've been feeling useful	0.02	0.07	0.21	0.01	0.00	0.04	− 0.14	0.03
9. I've been feeling close to other people	0.03	0.07	0.14	0.01	0.11	0.03	− 0.09	0.03
14. I've been feeling cheerful	0.08	0.07					− 0.09	0.03
10. I've been feeling confident	0.35	0.07						
3. I've been feeling relaxed	0.93	0.07	0.53	0.01	1.06	0.03	0.69	0.03

Item 1 “*I’ve been feeling optimistic about the future*” was not retained in our sample, but was retained in the Swedish, UK, and Australian cohorts, which why only 6 of the 7 item locations are shown in this Table for the Swedish and UK versions; and only 9 of the 10 items in the Australian version. Note that the Australian study reported on item locations for the original 14-item scale, not the final 10-item scale. The final 10-item version might have slightly different item locations, but they were not reported in the manuscript

**Fig. 3:**
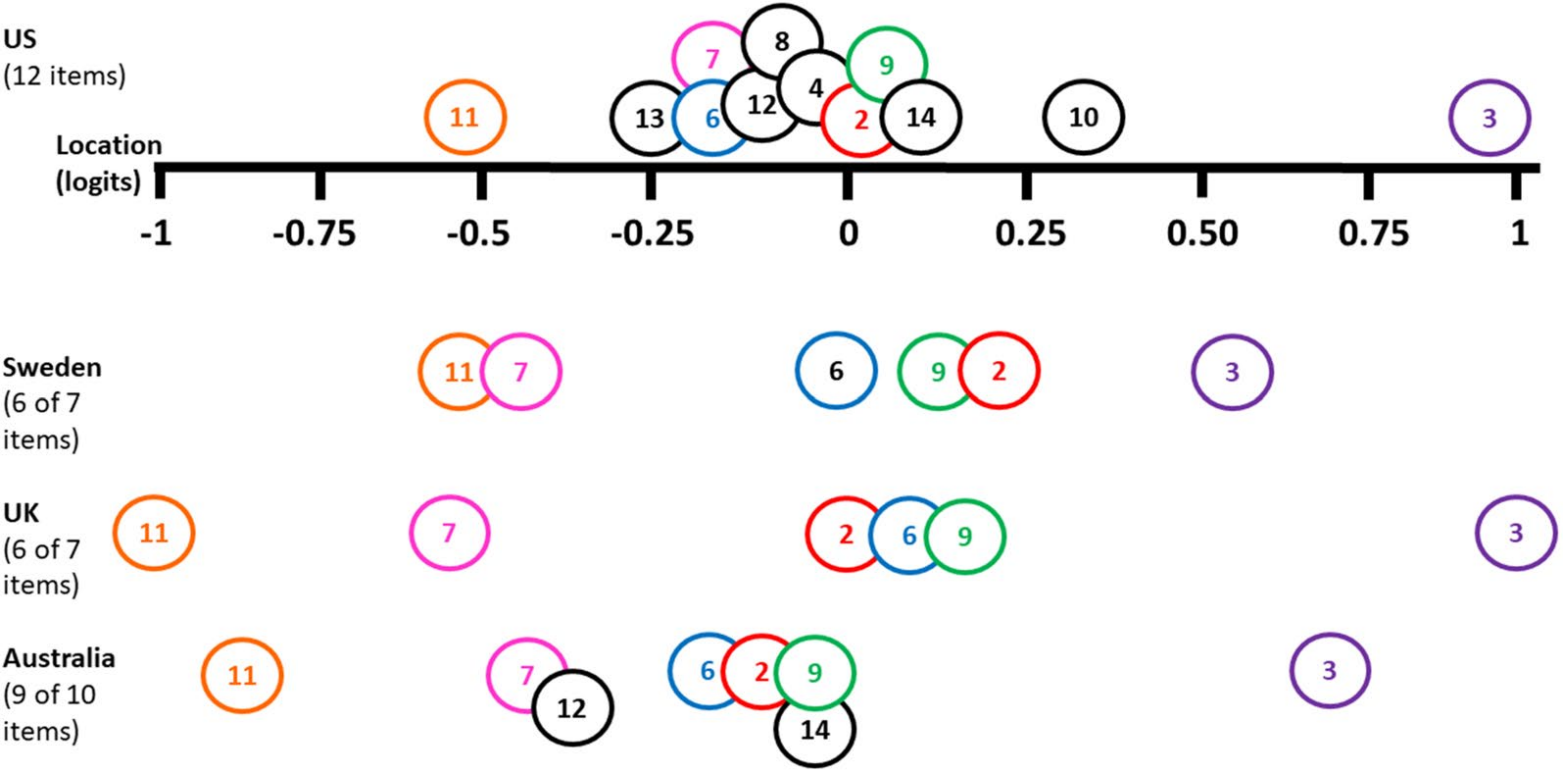
Item locations for our US cohort, compared to previously published Swedish, UK, and Australian cohorts. Item 1 “*I’ve been feeling optimistic about the future*” was not retained in our sample, but was retained in the Swedish, UK, and Australian cohorts, which is why only 6 of the 7 item locations are shown in this Figure for the Swedish and UK versions; and only 9 of the 10 items in the Australian version. The Australian study reported item locations for the original 14-item scale, not the final 10-item scale. The final 10-item version might have slightly different item locations, but they were not reported in the manuscript

Figure 3 displays the relative position of all items that the Swedish, UK, and Australian cohort has in common with the items reported in this manuscript. Item 11 "*I've been able to make up my own mind about things*” is the easiest item and item 3 “*I’ve been feeling relaxed*” is the hardest item across all cohorts [7, 8, 13]. Item 6 “*I’ve been dealing with problems well*” is relatively easier than item 2 “*I’ve been feeling useful*”, and item 2 is relatively easier than item 9 “*I’ve been feeling close to other people*” in the US and Australian cohorts, but this difficulty level order is slightly different in the Swedish cohort (order: items 6, 9, 2) and UK cohort (items 2, 6, 9) [7, 8, 13]. However, all items are located between − 0.19 and 0.21 logits. Item 7 “*I’ve been thinking clearly*” is situated around the same difficulty level range (between − 0.66 and 0.47 logits) in the Swedish, UK, and Australian cohorts but is rated more difficult in the US cohort (located at − 0.18 logits), which may also point to another interpretation of the concept “thinking clearly” in relation to culture in the US [7, 8, 13]. For example, this sentence may be rated more difficult to achieve if persons are thinking about “thinking clearly about what to do at work or achieving goals” in comparison to “thinking clearly in general, about daily (routine) activities”. Since we have not performed a qualitative study, we are unable to infer how our cohort has interpreted this sentence.

## Conclusions

The WEMWBS demonstrated good item fit and person fit in American community-dwelling adults. However, the items are too easy, which is a consistent finding across the majority of WEMWBS Rasch studies performed in different countries. Thus, including more difficult items in a next iteration of the scale could help solve the targeting.

## Supplementary Information

**Additional file 1.** Iteration table.

**Additional file 2.** Item threshold location.

**Additional file 3.** Frequency of scoring category responses for each item.

## Data Availability

The dataset(s) supporting the conclusions of this article is(are) available in the Data Repository for U of M (DRUM), https://doi.org/10.13020/jdfb-pn26.

## Abbreviations

WEMWBS: Warwick-Edinburgh Mental Well-Being Scale
SWEMWBS: Short Warwick Edinburgh Mental Well-Being Scale
RUMM: Rasch Unidimensional Measurement Model
UBACC: University of California, San Diego Brief Assessment of Capacity to Consent
PSR: Person Separation Reliability
DIF: Differential Item Functioning
PCAR: Principal Components Analysis of Residuals
