# Supplementary material for: Rasch validation of the Warwick-Edinburgh Mental Well-Being Scale (WEMWBS) in community-dwelling adults
Source: BMC Psychol. 2023 Feb 17;11:48. doi: 10.1186/s40359-023-01058-w (PMC9936469; doi:10.1186/s40359-023-01058-w)
Supplement: Supplementary file 1 — Additional file 1. Iteration table. [file 40359_2023_1058_MOESM1_ESM.docx]

**Additional file 1. Iteration Table**

| **Analysis** | **Items** | **Rating scale categories** | **Person mean (SD) logits** | **Mean error variance** | **Floor effect**  **n (%)** | **Ceiling effect**  **n (%)** | **Overall Chi-square (DF)**  ***p*-value** | **PSR** | **Items with disordered thresholds**  **(n)** | **Misfitting items**  **(n)** | **PCAR**  **Eigenvalue**  **1^st^ contrast**  **(%)** | **Misfitting persons**  **n (%)** |
| --- | --- | --- | --- | --- | --- | --- | --- | --- | --- | --- | --- | --- |
| Houghton *et al.* (2015)  Adults in Western Australia  (n=2005) | 10  (items 4, 5, 8, 10 deleted) | NA | NA | NA | NA | NA | 109.85  (90)  *p*=0.08 | 0.83 | 0 | 3  Item 1  (Res= 6.69) *p*=not available;  Item 3  (Res= 5.71)  *p*=not available; Item 13 (Res= 3.92) *p*=not available | NA | NA |
| Wicaksono *et al. (*2021)  Adults in Indonesia  (n=900) | 14 | 70 | 2.67  (1.56) | NA | 0 (0.00%) | 30**  (3.33%) | 205.50*  (*p*<0.01) | 0.89 | 0 | 0 | NA | 15**  (1.67%) |
| Stewart-Brown *et al*. (2009)  Adults in Scotland (16-74y)  (n=779) | 7  (items 4, 5, 8, 10, 12, 13, 14 deleted) | 35 | -0.48  (1.22) | NA | NA | NA | 64.70  (54)  *p*=0.15 | 0.85 | 0 | 0 | NA | NA |
| Bartram *et al.* (2013)  Veterinarians in the UK***  (n=500) | 7  (items 4, 5, 8, 10, 12,  13, 14 deleted) | 35 | 1.15  (1.56) | NA | NA | NA | 58.80  (56)  *p*=0.10 | 0.83 | 0 | 0 | NA | 72  (14.4%) |
| OUR DATA | | | | | | | | | | | | |
| Community-dwelling adults  (n= 553) | 14 | 70 | 1.93  (1.85) | 0.27 | 0  (0.00%) | 19  (3.44%) | 280.12  (126)  *p*<0.0001 | 0.92 | 0 | 2  Item 1 (Res=5.34, *p*<0.0001), Item 5 (Res=5.27, *p=*0.000009) | 1.99  (14.19%) | 19  (3.44%) |
| **Community-dwelling adults (n= 553)**  **Delete Items 1, 5** | **12**  **(items 1, 5 deleted)** | **60** | **2.17**  **(2.00)** | **0.35** | **0**  **(0.00%)** | **31**  **(5.60%)** | **179.84**  **(108)**  ***p=*0.00002** | **0.91** | **0** | **0** | **2.04**  **(16.97%)** | **15**  **(2.71%)** |
| **The analysis below was considered but not kept for final analysis** | | | | | | | | | | | | |
| Community-dwelling adults (n= 553)  Mimick other studies (delete 7 items 4, 5, 8, 10, 12, 13, 14) | 7 | 35 | 1.88  (1.71) | 0.46 | 0  (0.00%) | 26  (4.70%) | 103.50  (63)  *p*=0.001 | 0.82 | 0 | 0 | 1.86  (26.53%) | 3  (0.54%) |

***Legend:*** DF=Degrees of Freedom; PCAR=Principal Components Analysis of Residuals; PSR=Person Separation Reliability

* Wicaksono *et al.* (2021) reported the overall Chi-square as 20550, which is possibly a typing error; Res = Residual

** Wicaksono *et al. (*2021) deleted the misfitting persons as well as those that had a maximum score from the sample, thereby calculating the Rasch analysis on n=855

*** Bartram *et al.* (2013) The Rasch data set (n=500) was derived from two independent cross-sectional surveys of the veterinary profession (n = 8,829 and n = 1,796).
