# Supplementary material for: Rasch validation of the Warwick-Edinburgh Mental Well-Being Scale (WEMWBS) in community-dwelling adults
Source: BMC Psychol. 2023 Feb 17;11:48. doi: 10.1186/s40359-023-01058-w (PMC9936469; doi:10.1186/s40359-023-01058-w)
Supplement: Supplementary file 2 — Additional file 2. Item threshold location. [file 40359_2023_1058_MOESM2_ESM.docx]

**Additional file 2. Item threshold location**

| original item numbering | Location | Centralized Threshold 1 | SE 1 | Centralized Threshold 2 | SE 2 | Centralized  Threshold 3 | SE 3 | Centralized Threshold 4 | SE 4 |
| --- | --- | --- | --- | --- | --- | --- | --- | --- | --- |
| item 11 | -0.556 | -3.00 | 0.24 | -0.85 | 0.13 | 0.59 | 0.10 | 3.26 | 0.12 |
| item 13 | -0.245 | -2.40 | 0.22 | -1.03 | 0.13 | 0.46 | 0.10 | 2.97 | 0.12 |
| item 7 | -0.176 | -2.83 | 0.25 | -1.71 | 0.12 | 0.77 | 0.10 | 3.77 | 0.13 |
| item 6 | -0.176 | -4.88 | 0.20 | -1.21 | 0.11 | 1.27 | 0.10 | 4.82 | 0.17 |
| item 12 | -0.120 | -1.89 | 0.21 | -0.95 | 0.13 | 0.43 | 0.10 | 2.41 | 0.11 |
| item 8 | -0.109 | -3.78 | 0.20 | -1.00 | 0.11 | 0.84 | 0.10 | 3.94 | 0.14 |
| item 4 | -0.029 | -2.92 | 0.20 | -0.96 | 0.12 | 0.52 | 0.10 | 3.36 | 0.13 |
| item 2 | 0.019 | -2.65 | 0.21 | -1.27 | 0.12 | 0.36 | 0.10 | 3.56 | 0.13 |
| item 9 | 0.032 | -3.76 | 0.18 | -0.83 | 0.11 | 1.00 | 0.10 | 3.58 | 0.14 |
| item 14 | 0.080 | -2.54 | 0.22 | -1.62 | 0.11 | 0.71 | 0.10 | 3.44 | 0.13 |
| item 10 | 0.351 | -2.96 | 0.19 | -1.32 | 0.11 | 0.60 | 0.10 | 3.67 | 0.15 |
| item 3 | 0.929 | -3.53 | 0.15 | -1.39 | 0.10 | 0.77 | 0.10 | 4.15 | 0.19 |

***Legend:*** SE=Standard Error
