# Supplementary material for: Rasch validation of the Warwick-Edinburgh Mental Well-Being Scale (WEMWBS) in community-dwelling adults
Source: BMC Psychol. 2023 Feb 17;11:48. doi: 10.1186/s40359-023-01058-w (PMC9936469; doi:10.1186/s40359-023-01058-w)
Supplement: Supplementary file 3 — Additional file 3. Frequency of scoring category responses for each item. [file 40359_2023_1058_MOESM3_ESM.docx]

**Additional file 3. Frequency of scoring category responses for each item.**

| Item number | Item description | 0  None of the time | 1  Rarely | 2  Some of the time | 3  Often | 4  All of the time |
| --- | --- | --- | --- | --- | --- | --- |
| Item 11 | I've been able to make up my own mind about things. | 2 | 20 | 82 | 255 | 163 |
| Item 13 | I've been interested in new things. | 4 | 24 | 92 | 239 | 163 |
| Item 7 | I've been thinking clearly. | 2 | 19 | 124 | 271 | 106 |
| Item 6 | I've been dealing with problems well. | 1 | 31 | 159 | 270 | 61 |
| Item 12 | I've been feeling loved. | 7 | 26 | 87 | 209 | 193 |
| Item 8 | I've been feeling good about myself. | 2 | 37 | 124 | 258 | 101 |
| Item 4 | I've been feeling interested in other people. | 3 | 32 | 109 | 259 | 119 |
| Item 2 | I've been feeling useful. | 4 | 26 | 106 | 279 | 107 |
| Item 9 | I've been feeling close to other people. | 3 | 44 | 135 | 237 | 103 |
| Item 14 | I've been feeling cheerful. | 6 | 25 | 130 | 245 | 116 |
| Item 10 | I've been feeling confident. | 7 | 40 | 134 | 248 | 93 |
| Item 3 | I've been feeling relaxed. | 7 | 60 | 179 | 238 | 38 |
